# Pharmacist Intervention in Portuguese Older Adult Care

**DOI:** 10.3390/healthcare10101833

**Published:** 2022-09-22

**Authors:** Ana Rita Rodrigues, Edite Teixeira-Lemos, Filipa Mascarenhas-Melo, Luís Pedro Lemos, Victoria Bell

**Affiliations:** 1 Laboratory of Social Pharmacy and Public Health, Faculty of Pharmacy of the University of Coimbra, University of Coimbra, 3000-548 Coimbra, Portugal; 2 CERNAS-IPV Research Centre, Polytechnic Institute of Viseu, 3504-510 Viseu, Portugal; 3 Drug Development and Technology Laboratory, Faculty of Pharmacy of the University of Coimbra, University of Coimbra, 3000-548 Coimbra, Portugal; 4 REQUIMTE/LAQV, Group of Pharmaceutical Technology, Faculty of Pharmacy of the University of Coimbra, University of Coimbra, 3000-548 Coimbra, Portugal; 5 Nuclear Medicine Department, Centro Hospitalar e Universitário de Coimbra, 3000-548 Coimbra, Portugal

**Keywords:** pharmacists, older adults, medication management, healthy ageing, disease prevention

## Abstract

Healthy ageing has become one of the most significant challenges in a society with an increasing life expectancy. Older adults have a greater prevalence of chronic disease, with the need for multiple medications to appropriately control these issues. In addition to their health concerns, ageing individuals are prone to loneliness, dependence, and economic issues, which may affect their quality of life. Governments and health professionals worldwide have developed various strategies to promote active and healthy ageing to improve the quality of life of older adults. Pharmacists are highly qualified health professionals, easily accessible to the population, thus playing a pivotal role in medication management. Their proximity to the patient puts them in a unique position to provide education and training to improve therapeutic adherence and identify medication-related problems. This paper aims to address the importance of Portuguese community pharmacists in the medication management of older adults, emphasising their intervention in health promotion, patient education, medication-related problems, deprescription, dose administration aids, and medication review and reconciliation. We also discuss home delivery services and medication management in long-term care facilities.

## 1. Introduction

The increase in life expectancy and population ageing are currently significant societal concerns. The ageing process can be defined as an undergoing process of biological, psychological, and social change that occurs throughout each life cycle, starting before birth [1,2]. Although not consensual, a person aged 65 or older is referred to as an older adult, regardless of gender or physical condition [3]. According to the World Health Organization (WHO) [4], health status, autonomy, social participation, and level of independence may differ among each age group. These variations should be considered when developing policies and advisory programs for older adults.

According to data from the National Strategy for Active and Healthy Aging (ENEAS) [5], Portugal, likemost developed countries, has registered continuous demographic ageing in recent decades. An increase in the older adult population and longevity and the reductions in the younger population and birth rate are the main reasons for the current demographic situation.

In 2021, people aged 65 or more represented 23.4% of the population living in Portugal [6]. In the same year, the aging ratio (+65 per 100 < 15) was 182.1% compared to 27.3% in 1960 [7]. In the 2018–2020 triennium, life expectancy at birth was estimated at 78.07 years for men and 83.67 years for women [8]. OECD statistics regarding the health-related quality of life show that only 46% of the Portuguese population consider themselves in good health, a much lower percentage than the OECD average (69%). In 2019, Portugal had one of the lowest scores in perceived health status compared with other OECD countries [9], and 38% of the population had two or more chronic conditions [10]. In 2020, Eurostat concluded that although life expectancy at age 65 in Portugal was within the European Union (EU) average (EU, 21 years; Portugal, 21.6 years), expected healthy life years were below the EU average (EU, 9.8 years; Portugal 7.7 years). Furthermore, the discrepancy in expected healthy life years between sexes was larger in Portugal than in the EU. While in the EU, women had a higher number of healthy life years (10.1 vs 9.5 in men), in Portugal, the number of healthy life years for women was lower than for men (7.1 vs 8.4 years, respectively) [11]. These results justify the need for initiatives aimed at improving these indicators.

Increased demographic ageing, associated with the high prevalence of multimorbidity in older adults, strongly impacts society and threatens the sustainability of health care systems. In addition, physiological changes, multiple comorbidities, and a higher prevalence of chronic diseases can lead to polymedication in most elderly patients. Interventional strategies and policy frameworks should include multidisciplinary teams to increase health literacy, instigate active ageing and improve health outcomes [12].

Promoting active and healthy ageing can improve the well-being and the quality of life of older adults [5]. Community pharmacists, as highly qualified and accessible health professionals, play a pivotal role in promoting active and healthy ageing [13]. Acting at various levels and in articulation with other professionals, they contribute to achieving positive health outcomes. Pharmacist intervention to ensure the correct use of medicines and adherence to therapy helps to prevent drug interactions and adverse drug reactions, thus improving health and preventing further medication-related problems in older adults [14]. However, Portuguese health policies do not mention the importance of pharmacist intervention in the promotion of healthy ageing or the impact of their intervention in different care settings.

The present study aims to underline the importance of pharmacist intervention in Portuguese older adult care, namely, their involvement in medication management, long-term care institutions, and home care. We also address the role of pharmacists in improving drug-related knowledge and health literacy in older adults and their caregivers.

## 2. Methods

We conducted a brief review of the literature to assess the most current impact of pharmacists’ interventions on Portuguese older adult care patients. For this review, four databases that most commonly cite quality studies on pharmacist work were searched: PubMed/Medline, Google Scholar, and Medscape. Search terms incorporated both keywords and controlled vocabulary for the databases used. Example terms included “active-ageing”, “older adults”, “medicines related-problems”, and the “role of the pharmacist” in their titles, and abstracts were considered and scanned. The search terms were “pharmacist”, “older adult”, “medicine related-problems”, “medication management”, “healthy ageing”, “medication review”, “medication reconciliation”, “deprescription”, and “health literacy”. The search was restricted to the English language and included publications from 2018 to 2022 to encompass the most recent and widely viewed information. Letters to the editor, editorials, and conference proceedings were excluded. Review articles, systematic reviews, and meta-analyses were kept separately to be reviewed and discussed under the evidence of geriatric pharmacy care, tools used by pharmacists, and the role of pharmacists in geriatric care sections.

Governmental databases such as the WHO, Portuguese Directorate-General of Health (DGS—Direção-Geral da Saúde), International Pharmaceutical Federation (FIP), and Portuguese Order of Pharmacists were also analysed to include regulatory guidelines and laws.

A preliminary review of study titles and abstracts was conducted to exclude articles that did not meet the inclusion criteria: (1) subjects aged 65 years and older, (2) description of distinguishable pharmacist intervention, (3) utilisation of comparative design, and (4) measurement of patient-related health outcomes. A dual review was performed, and finally, a total of 157 publications, nonduplicated references, were evaluated and included as showing the current impact of pharmacists on the care of older adults. Data extraction was independently conducted by two of the authors.

## 3. Ageing and the Elderly

### 3.1. Disease Prevalence and Risk Factors in Older Adults

Ageing leads to organic changes, which result in a gradual decrease in all physiological functions, and the progressive and irreversible loss of the body’s ability to maintain homeostatic balance, thus leading to a greater propensity for pathologies in older adults [15].

Cardiovascular diseases, malignant neoplasms, chronic respiratory diseases, musculoskeletal diseases, diabetes [16], and neurological and psychiatric problems [17] such as depression [18] and anxiety [19] are among the most frequent pathologies in older patients. Multi-drug regimens are often necessary to manage these chronic conditions and, in most cases, inevitable [20]. Although no consensual understanding exists, polypharmacy is frequently defined as the simultaneous use of five or more medications [21]. Ageing patients with diminished cognitive and physical abilities may experience difficulties when confronted with complex multi-drug regimens. In these individuals, adherence may be compromised, and medication errors can occur, leading to increased adverse drug events (ADEs), unforeseen hospitalisations, negative health outcomes, and additional health costs [22].

Proper care and professional follow-up can help older adults to understand their medication better and contribute to their correct use in accordance with medical prescription [23], as addressed in a systematic review by Jaam et al. that showed the importance of pharmacist-led educational interventions in reducing medication errors [24].

According to data from Programa Nacional para a Saúde Das Pessoas Idosas (National Program for the Health of Older People) [1] in addition to genetic, biological, psychological, and individual factors, other external, environmental, behavioural, and social factors also influence ageing.

Loneliness, social isolation, physical and mental dependence, institutionalisation, financial problems, changes in the family structure, and an inappropriate housing environment are some of the main factors that can affect the health, autonomy, and quality of life of older adults [25,26]. Therefore, older adults benefit from and need more health care and social support, not only from health services but also from family and caregiving institutions [27].

### 3.2. Problems Associated with Medication Use in Older Adults

Polymedication is extremely common in older adults. The need for long-term multi-drug regimens to control or manage chronic diseases can trigger or aggravate drug-related problems and compromise the correct, effective, and safe use of medication [28,29].

A recent study shows that the overall prevalence of polymedication in the Portuguese population aged 65 and older was 36.9%, one of the highest among the 17 countries studied and above the reported average (32.1%) [20]. When addressing the 85-and-older age group, the overall prevalence of polypharmacy in the studied countries was 46.5%, with Portugal presenting the worst result (67.6%) [20].

The high prevalence of polymedication among older adults is often associated with adverse health outcomes. Increased risk of adverse drug reactions, drug interactions, therapeutic duplications, and potentially inappropriate medication use are among the most common problems [30].

According to the WHO, in 2002, approximately 50% of patients did not take their medication correctly [4]. More recent studies predicted that 16.4% to 61.4% of the population did not adhere to their prescribed therapy [31]. The incorrect use of medication is widespread in older adults and may result in drug inefficacy, increased frequency of adverse effects, drug interactions, and hospitalisation [32].

Successful chronic disease management depends on medical adherence. The degree to which the person’s behaviour corresponds with and follows professional health care recommendations is perceived as medication adherence and is considered essential for medicine efficacy [33].

Non-adherence is common in older adults and is a major risk factor in chronic disease management. Multiple factors can influence adherence. While higher health literacy, social status, and social support can contribute to medication adherence [34], it can be negatively impacted by other factors such as age, multi-drug regimens, multimorbidity, and cognitive impairment [35]. Non-adherence can be intentional, when the patient consciously decides not to take his medication, or non-intentional, when he forgets, does not understand the therapeutic regimen, or has a physical limitation [36]. Effective strategies to improve adherence can help manage medication safely and effectively [37].

As qualified and accessible health professionals, pharmacists are in an advantageous position to monitor polymedicated patients and provide pharmacotherapeutic counselling. Their intervention can help to improve medication adherence and prevent drug interactions and adverse events, contributing to the rational use of medicines [38]. Pharmacists can have a particular role in helping manage forgetfulness, difficulties in managing medication, and concerns with side effects, since these are the most frequent medication-nonadherence-related factors pointed out by older adults [39] Malham et al. [40] showed that pharmacist-led interventions could play a major role in the management of medicine-related-problems and economic issues in ambulatory care. These studies carry important information to discuss the role of the pharmacist in the healthcare system.

### 3.3. Social Responses for Older Adults

Population ageing has become a significant concern in developed countries. Social changes have altered family dynamics. Long working hours and demanding jobs have left family members unable to assist older adults, increasing the need for community-based support services for these individuals. Multidisciplinary support teams are essential to appropriately manage health conditions and ensure that older persons maintain a substantial degree of autonomy and social interaction [1].

In Portugal, social support services are legally regulated [41]. Different care options are available to help to ensure healthy ageing and provide social support. Older people and their families can choose from in-home support services, community centres, day-care centres, night centres, holiday and leisure centres, and residential care centres. Most older people want to stay in their own homes as they age. Moving may present mental and social challenges and can lead to physical and emotional distress [42]. If the elder has sufficient autonomy and has no life-threatening health problems, in-home support services, day- or night-care centres, or community centres may offer the needed care. However, full-time support systems are required when the older adult can no longer tend to their basic needs. Residential care homes provide temporary or permanent assistance to older adults who require daily support. In these institutions, full-time aid is available; health care needs are met; planned activities are offered; and social interaction is encouraged [43].

The above-mentioned social support responses aim to improve senior well-being and ensure their safety and quality of life. Nevertheless, assuring active and healthy ageing might delay the need for such support systems [44].

### 3.4. Strategies for Active and Healthy Ageing

While the worldwide increase in life expectancy might be seen as a triumph, global ageing can pose a serious challenge to modern society [4]. As we become older, we aspire to remain healthy and active. Healthy and active ageing not only enables individuals to continue to be autonomous in their activities of daily living and age with dignity but also helps society to maintain social and economic sustainability [4].

The health and well-being of older adults are affected by the physiological, psychological, social, and economic changes that occur with ageing. The problems resulting from these changes can interfere with the older adult’s ability to autonomously perform daily activities, making them more dependent and increasing the need for family or institutional support [45].

When evaluating the quality of life, health is generally considered as the most relevant factor; however, there are other aspects that should be considered and that are equally important to ensure wellbeing [46].

Social interaction has an undeniable importance in older adults’ lives. Contact with family and friends, community interaction, and leisure activities can provide comfort and tranquillity. Family ties promote a feeling of security and affection, which help to maintain the older adult’s emotional health [47]. The sharing of knowledge, values, and traditions with other generations can increase well-being, social integration, and recognition, thus preventing isolation. Social relationships can influence older adults’ quality of life as much as their physical health [47]. Recent studies have found that community pharmacist intervention can help to prevent social isolation and improve older adult care [48].

With ageing, motor coordination and cognitive abilities decline, rendering some daily activities difficult to perform comfortably and safely [49]. The risk of falls increases considerably with ageing and polypharmacy. Recent studies established that 28% to 35% of accidents involving older adults occur at home [50]. To prevent domestic accidents, living spaces must be adapted to the needs and limitations of older adults [5]. Poor lighting [51], the existence of slippery floors and non-resistant furniture, the lack of adequate ventilation, the absence of handrails on the stairs, and the existence of stairs with steps of different heights are some factors that may contribute to the occurrence of accidents [50,51,52,53,54]. Studies showed that community pharmacists can help implement fall prevention plans and increase patient awareness of the potential fall risk effects of some medications, thus reducing fall risk [55,56].

Recent studies showed that the use of technology may be advantageous in the care of the elderly. Electronic sensors, drop detectors, pressure mats, door monitors, smoke alarms are some examples of equipment that may improve the safety of older adults [57].

Since the beginning of the 21st century, the WHO has addressed the challenges related to global ageing [4,58]. This organisation defines active and healthy ageing as “the process of developing and maintaining the functional ability that enables well-being in older age” [47] and enhances rapid ageing and mental health as problems [59]. Ageing is not a linear procedure, and older adults may present diverse risk factors, such as morbidities, e.g., reduced mobility and chronic pain [59], and socioeconomic status [60], that may contribute to isolation. Loneliness and social isolation are risk factors for early mortality as much as smoking and a sedentary lifestyle [61], and these problems become more pressing in rural areas [62]. These risk factors must be considered when defining healthy ageing policies. The Portuguese government has recently addressed and promoted active and healthy ageing. The National Strategy for Active and Healthy Ageing (ENEAS) [5] was submitted in 2017, and the 2019–2023 Governmental Program foresaw an Action Plan for the Ageing Population, prioritising healthy ageing and the elderly’s quality of life [63].

In 2021, the RePEnSa network (Rede Portuguesa para Envelhecimento Saudável e Ativo) was created to encourage knowledge sharing between academia and the four leading consortiums dedicated to developing and supporting strategies to improve the elderly’s quality of life (Porto4Ageing; Ageing@Coimbra, Lisbon-AHA e Algarve Active Ageing) [64].

The main objectives of the aforementioned strategies are promoting health and improving the quality of life of older citizens.

Despite their qualifications and close contact with society, the potential role of pharmacists in promoting active and healthy ageing is neglected in these programs. Their integration into multidisciplinary teams can contribute to the rational use of medicines, improve health literacy, improve the social integration of older adults, and maintain the sustainability of health care systems [39,48,65].

## 4. The Importance of the Pharmacist in the Follow-Up of Older Adults

Demographic ageing has increased the number of patients with multiple co-existing pathologies, which are inevitably associated with polymedication [66,67]. Therefore, pharmacies are pivotal in providing health care to older adults.

Pharmacists, as primary caregivers with privileged access to older adults, are highly qualified and trusted professionals that play a significant role in medication management [68]. Their intervention can help to reduce non-adherence, drug interactions, and other medication-related problems. They can also provide medication review services and contribute to simplifying medication regimens [69]. As public health agents, pharmacists are responsible for improving health literacy and the rational use of medicines [38].

In Portugal, Decree-Law n.^0^ 307/2007 of 31 August [70] establishes the legal framework for community pharmacies. Ordinance No. 1429/2007 of 2 November [71] defines the pharmaceutical and other health and wellness promotion services that community pharmacies can provide. Domiciliary support, the administration of medicines and vaccines, early screening and testing, pharmaceutical care programs, health education programs, medication reconciliation and management, dose administration aids (DAAs), compounding, and emergency care are some services offered by Portuguese pharmacies. Disease prevention, the promotion of healthy lifestyles, and health literacy campaigns and programs are other valuable services undertaken by community pharmacists.

Interventions targeting older adults have been a major priority of the Portuguese National Health Plan (PNS) since 2004 [72]. Pharmaceutical activity should be articulated with the patients and their caregivers, as well as with other health professionals [73].

Some of the primary areas of pharmaceutical intervention in older-patient-oriented care may include: (i) promoting the correct, effective, and safe use of medicines while dispensing, providing medication review and reconciliation services, and through dose administration aid; (ii) promoting health literacy and informal caregiver training; (iii) medication management in long-term care facilities; (iv) domiciliary support; (v) identifying suspected at-risk patients; (vi) promoting active and healthy ageing.

Pharmaceutical activity is patient focused. The essential preventive and therapeutic services provided by pharmacists are crucial in maintaining/improving patients’ health and quality of life, reducing healthcare costs through therapeutical reconciliation and deprescription, and changing hospitalisation profiles (less frequent and less time in hospital care), since pharmacists can perform health management interventions across many disease states [74].

### 4.1. Promoting the Correct, Effective, and Safe Use of Medicines

The responsible use of medications is essential for the sustainability of healthcare systems. It bears benefits to individuals and society and provides economic gains. The global annual burden of medication-related problems is estimated to equal 42 billion USD [75].

To help overcome medication-related problems, the WHO [32,76] and many countries worldwide [77,78,79] have undertaken awareness campaigns to promote the rational use of medicines and encourage health professionals to develop public education programs to help ensure good health outcomes.

Among health professionals, pharmacists play an important role in this context. According to the current Portuguese legal framework, community pharmacies can implement “Pharmaceutical Care Programs”. In these programs, pharmacists review patient medication and evaluate their pharmacotherapeutic outcomes to improve medication use, reduce negative results, and enhance therapy safety and effectiveness [80,81,82]. The services rendered by pharmacies are in accordance with the Portuguese National Plan for the Safety of Patients 2015-2020 [83] and the Portuguese National Plan for the Safety of Patients 2021–2026 [84], which refer to the need to increase safety in the use of medication.

Pharmacotherapeutic follow-up, one of the most relevant areas of pharmaceutical care, is a patient-focused service that addresses health problems, health promotion, disease prevention, medication management, and health education [38]. This service may help improve patients’ quality of life, especially in older patients with complex therapeutic regimens due to simultaneous underlying health-related conditions [85].

Therefore, pharmacists have an active role in promoting the correct use of medicines, contributing to the success of the therapy, and reducing health-related costs [86].

In summary, Portuguese pharmacists can intervene in the following areas: (a) drug dispensation; (b) medication review; (c) medication reconciliation; and (d) dose administration aids.

#### 4.1.1. Drug Dispensation

When dispensing any medicine, the pharmacist must provide the patient or their caregiver with all the information necessary to ensure their correct use. The importance of medication adherence must be explained, and the patient should be informed of the risks associated with therapeutic non-compliance. The pharmacist should also be available to clarify any doubts that occur during treatment, provide follow-up services and refer the patient to a physician when necessary [87]. The pharmacist should briefly review the medication to detect any drug-related problems (such as interactions or duplication) when dispensing medicines. In addition, pharmacists represent an important formal support, as being seen as health professionals they give credibility and confidence to the client [68].

#### 4.1.2. Medication Review Service (RevM)

In 2018, Pharmaceutical Care Network Europe defined medication review as a “structured evaluation of patient’s medicines with the aim of optimising medicines use and improving health outcomes.” This entails detecting drug-related problems and recommending interventions [88]. Medication review thus correlates the Best Possible Medication History with the patient’s morbidities, preferences, or geriatric syndromes to produce a personalised medication strategy aligned with patient preferences and goals. This review can be performed whenever relevant, either in acute situations or periodically [89], to help patients to correctly manage, understand, and use their medicines [90].

Although RevM can be applied to all types of patients [89], those living in care homes, with complex therapeutic regimes, on medicines frequently related to medication errors, severely frail, or patients using potentially addictive medication should be prioritised [91]. RevM can also be performed at home and in long-term care facilities, always aiming to improve the quality of life of older adult patients.

Some situations can also prompt the need for a medication review, such as when patients are admitted to the hospital following a suspected adverse drug reaction, when they express concern with multiple medicine regimens, or when a health/care professional raises concerns regarding the patient’s capability to manage their medication [91].

When performing a medication review, the pharmacist must consider all the medicines the patient is using, including not only prescription and over-the-counter medication but also vitamin/mineral supplements and herbal/traditional medicines [91]. Furthermore, the following should be considered: the patient’s and caregiver’s views, knowledge, and questions concerning their medications; the safety of each drug; compliance with use; the patient’s risk factors for developing adverse drug events; and the potential requirement for monitoring [89].

Drug-related problems represent the most common reasons for hospital admissions and mortality in primary care [92]. It is estimated that if all precautions were taken, 30% to 55% of these problems would be prevented [93].

The medicine review service allows one to identify and help to solve issues with adherence to therapy, dosing, unintentional duplications, adverse reactions, drug interactions, and incorrect dosage, among others [94]. By assisting patients in managing their therapeutic regimens, pharmacists can help to reduce medication errors and contribute to increased adherence to therapy. As integrated members of multidisciplinary teams, they can easily share patient-related information with other health professionals and help improve patient care [94,95].

Worldwide, there are already many programmes for medication management where pharmacists play important roles. In Australia, pharmacists are included in a programme called “Home Medicines Review”, where an accredited pharmacist reviews medication use, helps to minimise adverse reactions, and improves health literacy [96]. Canada’s health system provides a pharmacist consultation called “MedsCheck”, where pharmacists interview the patients and review the patients’ prescribed and non-prescription medicines [97]. In England, pharmacists perform structured medication review in patients with complex therapeutical regimens [98], and they are also allowed to prescribe medicines as independent prescribers [99]. From an economic point of view, pharmacists have an impact on decreasing total health expenditures, decreasing unnecessary care, and decreasing societal costs, although further research is needed to support future payment models [100].

Portugal has already some health policies that highlight pharmacists’ skills, although no specific mention is made of these professionals [101,102]. Félix and colleagues estimated that community pharmacy services in Portugal provide a quality-of-life gain of 8.3%, resulting in savings of over 800 million EUR [38], despite the fact that most services performed by pharmacists are not remunerated and are paid by their users [101]. A study from Paiva et al. showed that of 88 polymedicated participants, 92.2% were willing to pay for a pharmacotherapy management service, such as medication review and pharmacotherapy follow-up [14]. Medication review remains to be fully implemented in Portugal, despite the existence of national and international guidelines. The lack of communication between pharmacists and other healthcare professionals, the fact that pharmacists do not have access to the full clinical information of patients, and a lack of support by the Portuguese health authorities are some of the reasons that may help to explain the delayed implementation of this pharmaceutical service [103].

#### 4.1.3. Medication Reconciliation Service (RecM)

RecM is a process that aims to obtain the Best Possible Medication History of a patient, gathering all medicines information provided by the patient, their family/caregiver, general practitioner, and community pharmacist. Therefore, the health professional, preferably the pharmacist, documents all medicines (prescribed and over the counter), supplements, and herbal products [104]. The main goal is to identify and correct drug discrepancies such as omissions, duplications, or dosing errors [89], updating the patient’s information through all health care services [104].

Both RecM and RevM are processes with the purpose of increasing not only patient safety but also therapeutic effectiveness and efficiency. While RevM is a structured evaluation of a patient’s medications with the stated goal of detecting and solving drug-related problems, RecM is defined as the formal process of collecting a complete and accurate list of each patient ’s current medications with the objective of detecting and solving discrepancies. Therefore, medication reconciliation can be considered an automatic pre-requisite for a medication review, as the medication list should be as accurate as possible before it can be critically appraised.

According to Directorate-General of Health (DGS—Direção-Geral da Saúde) Standard 018/2016 [102], “medication reconciliation is a process that helps to keep each patient’s list of medication up-to-date, as well as other important information, including adverse reactions to medications and allergies, avoiding discrepancies between their usual medication and the medication instituted at each time of care transition”. In 2021, the International Pharmaceutical Federation (FIP) launched a toolkit for pharmacists regarding medicines reconciliation [105]. In this document, aligned with international evidence, the FIP demonstrates the economic impact of RecM and supplies key elements to provide this service (templates, tools, and step-by-step process).

This service is patient-centred [106] and involves a multidisciplinary team. It is carried out mainly during the transition of care, such as admission, hospital discharge, and after transfers between health care institutions, thus differing from the medication review service [102]. Medication reconciliation services may prevent 75–80% of clinically relevant medication variances in patient care [104].

Pharmacists may play a significant role in RecM. Their technical and scientific skills ensure that an up-to-date therapeutic regimen is maintained for each patient, enabling the detection of discrepancies; the reduction in possible medication errors, such as omissions or duplications of therapy; and the detection of problems when transitions of care occur. The pharmacist should promote information sharing regarding the medication and its discrepancies with other health professionals who follow the older adult so that they can correct them together [107]. A systematic review [104] concluded that pharmacy-led medication reconciliation reduced by 68% the proportion of patients with at least one discrepancy and by 88% the number of medication discrepancies.

Portugal has taken some steps toward implementing medication reconciliation, mainly in medical institutions such as hospital settings [108]. According to Oliveira et al. [109], pharmacist-led medication reconciliation in a psychiatric hospital in Portugal reported that one in three discrepancies required further clarifications and that 80% of them were unintentional. Another study by Costa e Silva et al. [110] in a Portuguese Internal Medicine Department reported that 95.7% of the discrepancies were unintentional and that almost half of them (49.1%) were not documented. Yet, as with most EU countries, this service is implemented with unclear and heterogeneous rules [110]. Several obstacles to the successful implementation of RecM have been identified. For this service to be successful, it is essential to increase the presence of pharmacists in multidisciplinary teams. An effective IT system that allows healthcare professionals to record information during the various phases of patient care transition and a proper articulation between the hospital and health centres, as well as between the hospital and community pharmacies, isalso needed [106]. It is also necessary to increase the health literacy of patients (the centre of this intervention), their caregivers, and the institutions that receive the older adult after hospital discharge [107]. To achieve better outcomes, it is also essential to promote clinical training amongst pharmacists [82] and further comprehensive research.

#### 4.1.4. Dose Administration Aids (DAAs) 

Dose administration aids are personalised medication devices that organise oral solid dosage forms according to the prescribed dose and schedule [111]. Tablets and capsules are repacked into individually sealed compartments according to how they must be taken [112]. These devices help simplify medication management and improve adherence in polymedicated patients suffering from chronic diseases [112,113]. DAAs can help reduce medication-related hospitalisations and, consequently, the costs associated with drug-related problems [114]. There are many dose administration aid devices, and the pharmacist has to choose which one is appropriate for the patient’s therapeutic regimen, using their expertise and considering the patient’s needs. With this in mind, the pharmacist should perform a medication review before implementing a DAA [112].

Despite the above-mentioned advantages, there are few studies concerning the effectiveness of DAAs in therapeutical adherence [115] and their costs compared to not using this service over extended periods. In addition, not all medicines are stable enough to be included in these devices, whereas in others, this information is not clear in product labelling. Therefore, the pharmacist should evaluate the stability of each dosage form (which medicines and how long they can be stored in the DAA) [112] to ensure optimal patient care. 

In Portugal, DAAs are regulated through Ordinance No. 455-A/2010 of 30 June [116], and pharmacists must follow the General Standard for Dose Administration Aids [117]. This document contains the safety rules that must be followed and the selection criteria that must be observed when implementing this service, such as flowcharts of DAA implementation and warnings regarding all steps of the process. A study among Portuguese pharmacies [115] analysed DAA characteristics, preferences, and impact on patients’ life. It concluded that most patients indicated fewer drug-related problems associated with forgetfulness and incorrect drug use, contributing to greater therapeutic adherence. 

This area of pharmaceutical intervention contributes to the promotion of the correct, effective, and safe use of medicines in older adults, facilitates adherence to therapy, and minimises medication errors, thus improving older adults’ quality of life.

**Figure 1:**
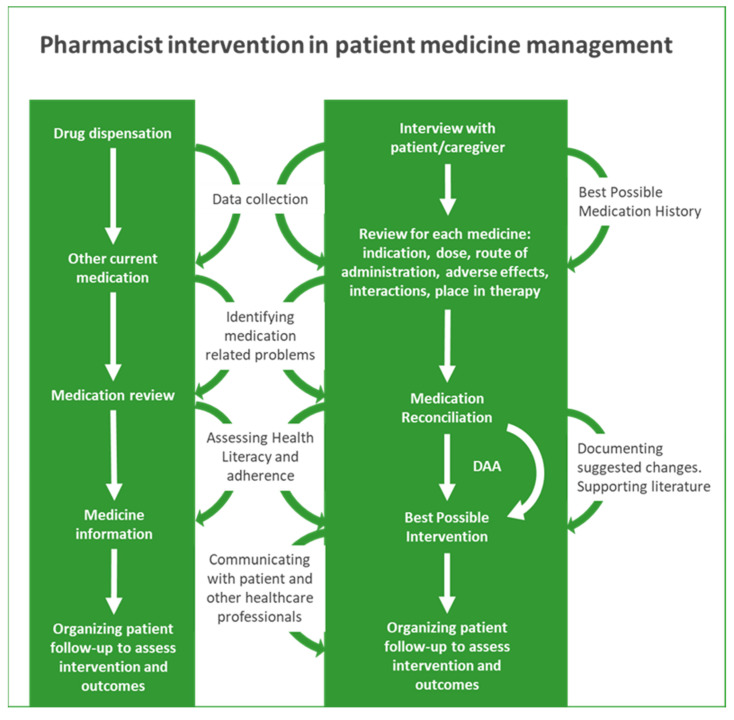
Pharmacist intervention in patient medication management.

Figure 1 summarises possible/desired pharmacist interventions in older adult medication management.

## 5. Emergent Challenges

Considering the topics discussed above, increasing the participation of pharmacists within the healthcare system can improve positive outcomes for patients and the healthcare system. Globally, emergent pharmaceutical services are developing to complement traditional services such as compounding and dispensing prescription medicines [118]. Some of these new pharmaceutical services are reimbursed by national governments [119]. Community pharmacies are easily accessible to patients, placing the pharmacist in a privileged position to provide healthcare services to the population [118]. Less medicine-related problems mean better clinical outcomes. In Portugal, despite recent guidelines from the DGS and the Portuguese Order of Pharmacists, improvements can be made regarding the implementation of these new and invaluable pharmaceutical care services.

### 5.1. Deprescription

Taking multiple medications is within treatment guidelines (such as hypertension or diabetes) to achieve specific treatment goals. However, the presence of multiple comorbidities can lead to a “prescribing cascade” [120]. As mentioned, polypharmacy is a significant concern in older adults. It is associated not only with drug-related problems such as interactions and toxicity but also with non-adherence due to complex therapeutic regimens. Polymedicated patients have more medication costs, have an increased risk of falls, and are likely to be hospitalised more frequently [120].

Deprescription is an intentionally planned and supervised intervention to withdraw a medicine or reduce its dose when it is no longer beneficial [121]. Anticholinergic medications, oral antidiabetics, nonsteroidal anti-inflammatory drugs (NSAIDs), and benzodiazepines are among the most deprescribed therapeutical classes. The main goals of deprescription are to prevent drug-related problems, reduce costs, and simplify therapeutic regimens [120]. Deprescription is only possible after medication reconciliation or medication review sessions identify useless, dangerous, or inappropriate medicines [122]. Therefore, it usually occurs following an adverse drug reaction or in response to polypharmacy, prescribing cascades, or at the end of life/palliative care [121]. Hospital and community pharmacists are qualified and can actively participate in this process.

### 5.2. Update for Review and Reconciliation of Medication

In 2021, the International Pharmaceutical Federation (FIP) published a toolkit for pharmacists regarding medicines reconciliation (RecM) [105], motivated by a WHO initiative to reduce severe and avoidable medication-associated harm by 50% in 2022. This toolkit provides a structured protocol for pharmacist-led medication reconciliation as a step to promote patient safety by reducing the risk of medication errors and potential harm in the transition of care. It also explores the clinical and economic impact of RecM and the tools needed to perform this service.

Considering national and international guidelines regarding medication safety and polymedication, the Portuguese Order of Pharmacists published, in 2021, the “Medicines Review Guideline” [123], describing each step of the process, the eligibility conditions, responsibilities, and indicators. Although it can be performed for patients of any age, this guideline considers older adults the main target population for Medication Review (RevM).

In 2022, the FIP launched an updated version of the toolkit renamed “Medication review and medicines use review: A toolkit for pharmacists” [124]. This new toolkit introduces medicines use review (MUR) as a subtype of RevM in which pharmacists partner with patients, taking their preferences and literacy into consideration, with the goal of improving adherence. RevM, on the other hand, is a more comprehensive and ambitious service [125], depending on a health team that assesses the patient’s therapeutic regimen to conceive an optimised plan regarding clinical, social, and economic outcomes (including adherence). 

Pharmacists, as medication experts, play a crucial role in the health system. These pharmaceutical services can help to reduce adverse events and direct and indirect medicine costs [124]. The FIP also suggested a suitable remuneration scheme to ensure the quality and sustainability of the services, and the proper compensation for the pharmacist’s expertise. This document also emphasises that pharmacist-led services can help to improve therapeutic outcomes, deprescription, and preventable adverse drug effects, thus increasing patient safety. The FIP also considers older adults to be the age group that benefits the most from these services. Despite all recommendations for pharmacist-led services, in Portugal, there is still room for improvement in health policies [126]. According to Policarpo et al. [68], pharmacy services fit the existing needs, although 29% of the participants believed there could be more services available in pharmacies that are being provided in other health care facilities.

### 5.3. Promotion of Health Literacy in Older Adults and Training for Informal Careers

Present-day health challenges are increasingly complex. Cultural diversity and social inequalities determine the existence of population groups with specific needs that require targeted approaches to solve their health problems.

A possible definition of health literacy is the “ability of an individual to obtain and translate knowledge and information in order to maintain and improve health in a way that is appropriate to the individual and system contexts” [127]. This definition contemplates the general issues acknowledged in the recent literature as important on this matter: (1) knowledge of health, healthcare, and health systems; (2) processing and using the information in various formats concerning health and healthcare; (3) ability to maintain health through self-management and working in partnerships with health providers [127]. The ability to obtain, process, and understand health information can condition behaviours, decisions, and the use of resources in this area. 

Therefore, health literacy is important and contributes to disease prevention and health promotion. Low levels of health literacy have been linked to an increase in hospitalisations, increased use of emergency services, and the misuse of health technologies [128,129]. These facts demonstrate the impact of health literacy not only on the health and well-being of the population but also on the sustainability of health systems [130]. On the contrary, individuals with higher levels of health literacy show improved adhesion to treatment and can manage their health [131].

Improving health literacy is currently a public health challenge and a priority for governments and health agencies [132]. Empowering users with the necessary know-how to understand and exercise greater control over their health is undoubtedly one of the main objectives of health education [131]. 

Health professionals, who are directly involved in care, need to develop skills that enable them to help the population to increase their level of health literacy. These professionals should analyse and interpret health or disease situations, identifying their determinants to conceive and implement actions that contribute to resolving the issue at hand. These actions should then be assessed to evaluate their effectiveness.

Health promotion interventions should not only be based on scientific evidence but also be guided by ethical and deontological principles. Appropriate communication techniques should be used to correctly convey the desired information and ensure it is appropriately understood. Thus, health professionals should develop strategies to modify individual and collective attitudes and behaviours to improve health outcomes.

In Portugal, health literacy is addressed in several official documents. The National Program for Education for Health, the Literacy and Self-Care (Programa Nacional de Educação para a Saúde, Literacia e Autocuidados) [133], the National Strategy for Active and Healthy Aging (ENEAS—Estratégia Nacional Para o Envelhecimento Ativo e Saudável) 2017–2025 [5], the Health Literacy Action Plan 2019–2021 (Plano de Ação Para a Literacia Em Saúde) [134], and the Good Practices of Health Literacy—Training of Health Professionals (Manual De Boas Práticas Literacia Em Saúde) [135] are some examples.

As mentioned, pharmacists are highly qualified and easily accessible health professionals, and according to Portuguese law, they are also public health agents [136]. Therefore, they play an important role in health education and promotion, contributing to improvements in health literacy and in individual and collective well-being.

Actions to promote health literacy and improve attitudes toward health should include older adults as well as their caregivers [137]. Informal caregivers were legally recognised by Portuguese law in 2019 [138], ensuring they receive financial and social support and an appropriate articulation with all health services. As a qualified healthcare professional, the pharmacist plays an important part in assisting and guiding caregivers in providing the best possible care to older adults [5].

### 5.4. Pharmacy Support to Long-Term Care Facilities (LTCF)

According to official data, between 1998 and 2018, there was a 105% increase in the number of long-term residential care facilities in Portugal [139]. In 2018, 2507 long-term residential care facilities existed in Portugal, with a capacity of 100518 users [139].

Transitioning into care facilities is not always easy. To adapt to the new environment, the older person must change their daily habits, and individuality and privacy need to be adjusted.

Although the LTCF population is heterogeneous, most institutionalised patients are chronically ill, polymedicated [140], and predominantly not autonomous enough to manage their medication. For this reason, LTCFs must guarantee the acquisition, storage, and distribution of prescribed medicines and ensure their correct administration. We believe that pharmacists are indispensable in this process. 

Although LTCFs are legally regulated, they are not required to possess pharmaceutical services [141]. In our opinion, this legal loophole is a cause for concern. Including pharmacists in long-term care multidisciplinary teams can ensure that all procedures comply with all medication-related legal standards. This significantly improves drug management in these institutions, with positive results for the health and well-being of institutionalised residents. However, the pharmacist’s intervention in LTCFs can be more comprehensive. As highly qualified health professionals with unique knowledge and training regarding medicines, they can detect potential and existing medication errors, drug interactions and adverse effects. Through medication review or medicines use review, pharmacists can help drug-related problems to be avoided or mitigated, reducing medication errors and improving health outcomes and the rational use of drugs [142].

In 2014, the Portuguese Institute of Quality (IPQ) issued a recommendation for “Medication Management in Residential Structures for The Healthy (ERPI)” [143]. This document acknowledges that in Portugal, there is a “scarcity of specific legislation defining procedures and quality standards regarding the prescription, acquisition, storage, distribution, administration and use of medicines in ERPI” [143]. It also states that the institutionalised elderly need the same quality guarantees regarding medication use as community patients [140].

The document also stresses the need for appropriate procedures to guarantee that patients “receive their medicines correctly, effectively and safely”. These should include personnel with “competencies in the management of the medicinal product, within their professional tasks, to ensure that patients enjoy a maximum therapeutic benefit resulting from drug treatment” [143].

Regarding the acquisition and dispensation of medicinal products, the IPQ recommends that “it should be ensured that a legally qualified professional evaluates the prescription of patients and provides all the information necessary for the correct, effective and safe use of the drug, in order to prevent negative results associated with medication” [143]. Whenever a new patient is admitted, a complete and up-to-date record of their medication must be prepared, and medication reconciliation should be performed to reduce errors and contribute to the health and well-being of the patient [144].

In long-term care facilities, all legal requirements regarding the acquisition and storage of medicinal products should be observed, ensuring storage conditions, avoiding contamination, and securing adequate turnover [145]. According to Portuguese law, all stages of the medicine supply chain (production, distribution, and retail sale) must have a pharmacist as the responsible person [146]. Although long-term care facilities are not legally obliged to have a pharmacist, we believe that by doing so, medicine acquisition, storage, and distribution would be improved, and most importantly, medicine-related problems could be avoided in these facilities [45].

The role of pharmacists in medication management is recognised in several countries. In the United States of America (USA) [147], the United Kingdom [148,149,150], Australia [151], and Canada [152], their role has been defined and is accepted as necessary. Pharmacists who are clinical experts and perform medication regimen reviews are referred to as consultant pharmacists and as senior care pharmacists when they specialise in older adult medication management [147]. Moreover, pharmacists can inform and educate patients about their treatment. Therefore, it is important to reinforce the need for a pharmacist responsible for the medication circuit in each long-term care facility, depending on their size.

### 5.5. Pharmacy Support for the Older Adults

According to official data, the number of older adults living alone in Portugal has escalated in recent years [153]. Between 2018 and 2021, the percentage of single-person Portuguese households aged 65 and older increased from 54.1% to 68.2% [153]. Living alone can lead to social isolation and loneliness, which have been linked to a higher risk for serious health problems, such as high blood pressure, heart disease, obesity, and physical and cognitive decline [154].

Home delivery is one of the many services that pharmacies can provide, according to Portuguese legislation [155]. This service enables older patients with mobility issues or without family/caregiver support to receive their medicines at home, delivered by a qualified health professional. Due to the high prevalence of morbidity and mortality associated with the use of medications in older adults, it is essential to monitor their medication, ensure adherence, and help them with any related problems. Of the 2918 pharmacies in PortugaL [156], 2803 offer home delivery to patients [157].

When providing this service, pharmacists always offer professional information and advice regarding medication. They can also evaluate various biochemical and physiological parameters when necessary (glycemia and blood pressure, among others). Pharmacists can also help patients/caregivers better understand their therapeutic regimens, increasing adherence, preventing drug-related problems, and promoting health literacy [155].

## 6. Conclusions

Ageing is an irreversible biological process associated with functional, psychological, and social changes. Older adults have a greater prevalence of chronic disease, with the need for multiple medications to appropriately control these issues. Active and healthy ageing is desirable, as it can help older adults maintain their independence and prevent or delay the onset of many diseases.

As extensively demonstrated above, Portuguese pharmacists are highly qualified professionals that must have a strategic role in the health care of elderly patients. Their intervention in long-term care facilities and communities constitutes an essential contribution to health care.

Medication use in older adults requires additional attention, since a large percentage of this population is polymedicated and at an increased risk of drug interactions, adverse reactions, and poor adherence to therapy.

Pharmacists are in an ideal position to monitor these patients and help to identify drug-related problems and patients at risk of underlying chronic diseases and nutritional, functional, and social issues, such as loneliness and lack of support.

Drug dispensation, medication review and reconciliation, and drug administration aids are examples of pharmaceutical services that can help to prevent health and medication-related problems by increasing adherence to therapy and improving the quality of life of older patients. Deprescription, the creation of new guidelines for medicine reconciliation, and the implementation of health literacy programs present challenges in which pharmacists can play a fundamental part.

However, unlike in other countries, there is a lack of formal recognition of the importance of the role of Portuguese pharmacists in long-term care facilities. Pharmacists can ensure safe, correct, and effective access to medication for each institutionalised older adult. Their presence in these facilities must, therefore, be considered a necessity.

Pharmaceutical home care is another essential pharmaceutical service. This service can go far beyond delivering medication at home; it can improve access to information on medicines and their correct use, thereby improving adherence and reducing the prevalence and severity of drug-related health problems.

Finally, Portuguese pharmacists and their representative associations must undertake efforts to promote these pharmaceutical services and encourage the inclusion of pharmacists within multidisciplinary teams, where they can make critical contributions by transferring information between all professionals and bridging the gap between professionals and patients. It is time to consider the strategic role Portuguese pharmacists can have in improving the health care of older patients.

## Data Availability

Not applicable.
